# Isolated tricuspid Libman–Sacks endocarditis in a patient with systemic lupus erythematosus and antiphospholipid syndrome: case report

**DOI:** 10.1093/ehjcr/ytaf493

**Published:** 2025-10-03

**Authors:** Gamaliel Alejandro Velasquez-Orozco, Yancy Yuliana Erazo-Dorado, Juan Carlos Rivera Guerrero, Felipe Israel Lopez Trejo, Elias Noel Andrade-Cuellar

**Affiliations:** Department of Cardiology, National Medical Center ‘November 20th’, ISSSTE, Mexico City C.P. 03104, Mexico; Department of Cardiology, National Medical Center ‘November 20th’, ISSSTE, Mexico City C.P. 03104, Mexico; Department of Cardiology, National Medical Center ‘November 20th’, ISSSTE, Mexico City C.P. 03104, Mexico; Department of Cardiology, National Medical Center ‘November 20th’, ISSSTE, Mexico City C.P. 03104, Mexico; Department of Cardiology, National Medical Center ‘November 20th’, ISSSTE, Mexico City C.P. 03104, Mexico; Universidad Nacional Autónoma de México, Av. Felix Cuevas #540, Col. Del Valle Del. Benito Juarez, Mexico City C.P. 03100, Mexico; Cardiac Electrophysiology, National Medical Center ‘November 20th’, ISSSTE, Mexico City C.P. 03104, Mexico

**Keywords:** Libman–Sacks endocarditis, Systemic lupus erythematosus, Antiphospholipid syndrome, Tricuspid valve, Massive tricuspid regurgitation, Three-dimensional vena-contracta area, Subarachnoid haemorrhage, Bioprosthesis, Case report

## Abstract

**Background:**

Libman–Sacks endocarditis (LSE) is an immune-mediated, culture-negative valvulopathy complicating systemic lupus erythematosus (SLE) and often amplified by antiphospholipid syndrome (APS). Although classically left-sided, isolated tricuspid involvement is rare. Advanced three-dimensional (3-D) quantification refines tricuspid regurgitation (TR) grading and informs surgical timing.

**Case summary:**

A 41-year-old woman with 2-year SLE and triple-positive secondary APS presented with 2 weeks of fever, migratory arthralgia, and a small non-traumatic subarachnoid haemorrhage (SAH). Serial blood cultures were negative. Transthoracic echocardiography showed multiple heterogeneous vegetations (largest 20 × 12 mm) on all tricuspid leaflets; 2-D PISA-EROA was 30 mm², while 3-D vena-contracta area (VCA) measured 0.95 cm², indicating massive TR. Cardiac computed tomography corroborated leaflet thickening and poor coaptation; transoesophageal echocardiography was deferred owing to recent SAH. A Heart Team favoured LSE over infective endocarditis. After high-dose corticosteroids for an SLE flare, surgery was deferred 21 days post-SAH and tricuspid valve replacement with a 31-mm bovine pericardial bioprosthesis was performed. Pathology confirmed sterile platelet–fibrin vegetations. Post-operatively, she received warfarin (target INR 2.5–3.5; heparin bridge), rituximab, hydroxychloroquine, and tapering prednisone. At 3 months, she was asymptomatic (NYHA I) with a competent prosthesis, normal right-sided dimensions, and improved lupus biomarkers.

**Discussion:**

This case highlights (i) the need to consider LSE in culture-negative right-sided endocarditis among SLE/APS patients; (ii) the clinical utility of 3-D VCA to reconcile discrepant 2-D measures and substantiate surgical indication; (iii) peri-operative strategies after recent SAH (timing and anticoagulation); and (iv) rationale for a bioprosthesis in the low-flow tricuspid position given thrombogenicity of mechanical valves, alongside lifelong vitamin K antagonist therapy mandated by APS. Early multimodality imaging, Heart Team decision-making, timely surgery, and optimized immunomodulation can yield excellent outcomes.

Learning points

- In systemic lupus erythematosus/antiphospholipid syndrome (APS), isolated tricuspid Libman–Sacks endocarditis should be suspected when right-sided vegetations with negative blood cultures and severe regurgitation are present.
- In the tricuspid position, the choice of a bioprosthesis reduces thrombotic complications compared with mechanical valves, even in APS patients requiring lifelong anticoagulation.

## Introduction

Libman–Sacks endocarditis (LSE) is the most prevalent cardiac manifestation of systemic lupus erythematosus (SLE). Sterile platelet–fibrin vegetations develop on damaged endothelium and undergo immune-complex deposition and fibrous organization that ultimately distort valvular architecture and function.^1–3^ Although the mitral and aortic valves are most often affected, isolated tricuspid involvement is exceptionally rare, with scattered case reports over the last three decades.^4–9^ The coexistence of antiphospholipid syndrome (APS) is a key accelerator of valvular injury—promoting leaflet thickening, sterile vegetations, embolism, and faster progression to haemodynamically significant regurgitation.^10^

Echocardiographic surveys of unselected SLE cohorts report valvular abnormalities in up to 50%, yet true LSE vegetations are visualized in only 4%–11% on transthoracic imaging; sensitivity improves with transoesophageal and three-dimensional techniques.^1–3^ Clinically, most lesions remain silent, but a meaningful subset—particularly those with high-titre antiphospholipid antibodies—develops severe regurgitation or thromboembolic complications that may warrant surgery.^1,10^ In contemporary practice, advanced quantification such as three-dimensional vena-contracta area (VCA) helps reconcile discrepant two-dimensional measures and better reflects the actual regurgitant burden, especially in noncircular or dynamic orifices typical of tricuspid regurgitation.^4,11^

We present a rare case of isolated tricuspid LSE producing massive regurgitation, outline multidisciplinary decision-making and perioperative strategy, and integrate updated evidence on imaging, APS-related risk, and indications for intervention.

## Summary figure

**Figure:**
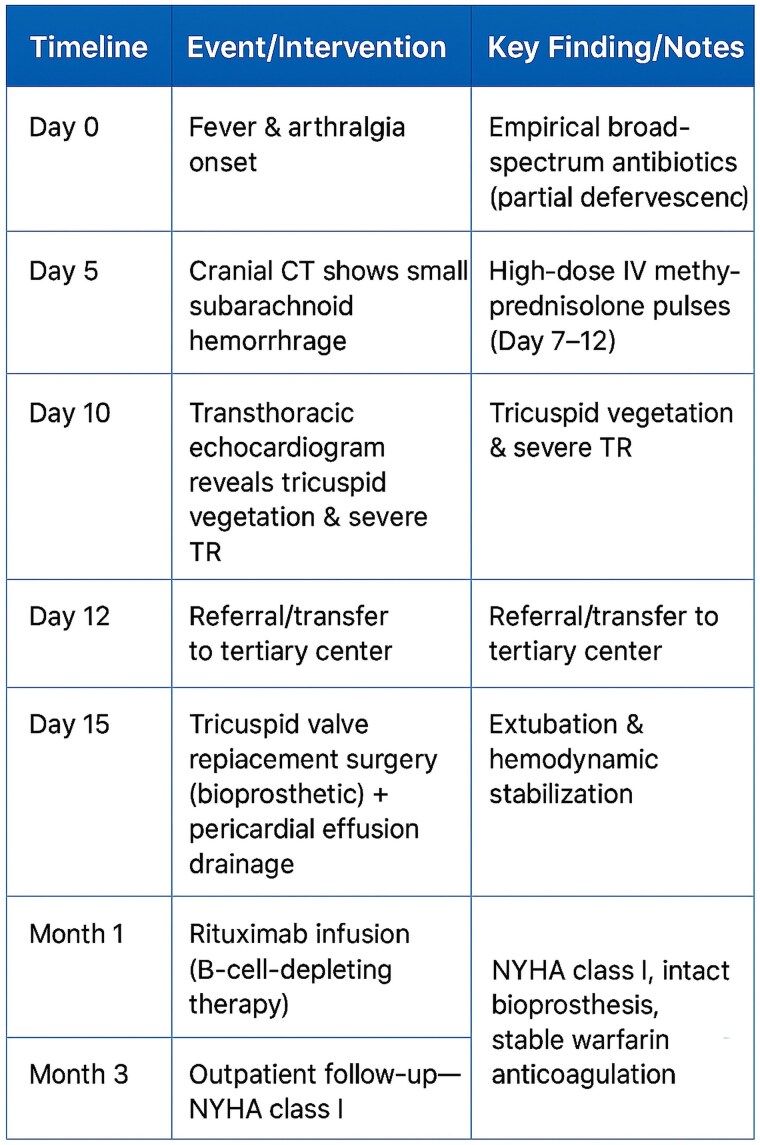


## Case presentation

A 41-year-old woman with a 2-year history of SLE and triple-positive secondary APS (on azathioprine) was admitted after two weeks of intermittent fever, migratory arthralgia, and myalgia. Broad-spectrum antibiotics given elsewhere failed to achieve defervescence. On Day 7, she developed headaches; cranial computed tomography (CT) demonstrated a small non-traumatic subarachnoid haemorrhage (SAH).

Initial laboratory work-up revealed bicytopenia attributable to active SLE. Pulse methylprednisolone (1 g day⁻¹ for 3s days) was started, yet fevers persisted and repeated blood cultures were negative. Transthoracic echocardiography (TTE) identified multiple heterogeneous masses on all three tricuspid leaflets—the largest 20 × 12 mm—with an effective regurgitant orifice area (EROA) by 2-D PISA of 30 mm² and a regurgitant volume of 26 mL, suggestive of at least moderate–severe regurgitation.

**Figure 1:**
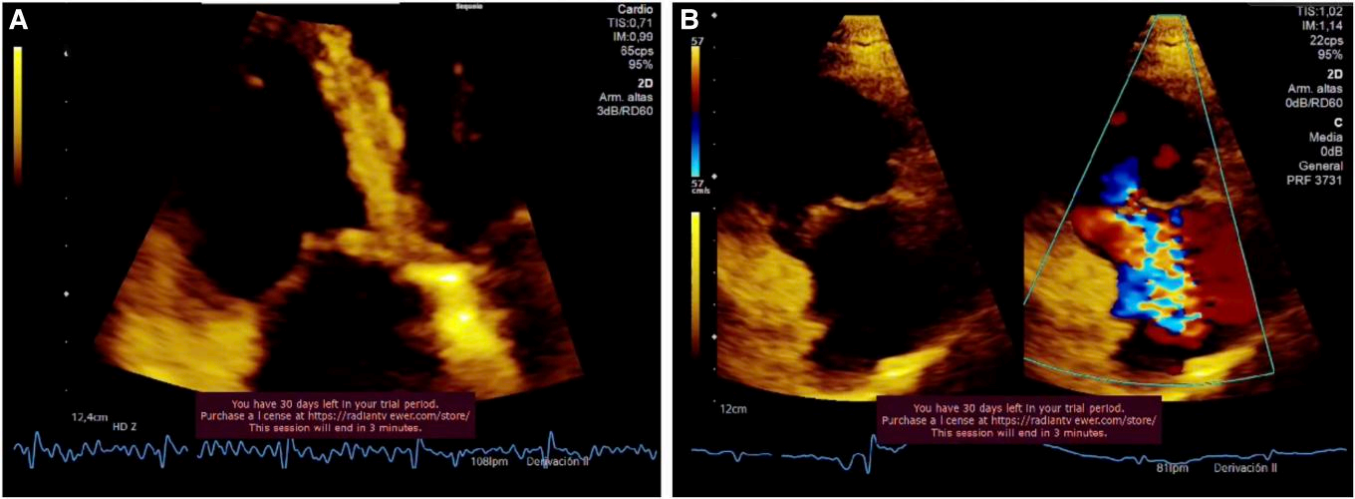
Transthoracic echocardiogram with a four-chamber view focusing on the right ventricle (*A*) and the right ventricular inflow tract (*B*). Thickening of the septal leaflet of the tricuspid valve with severe regurgitation is observed. RV, right ventricle.

**Figure 2:**
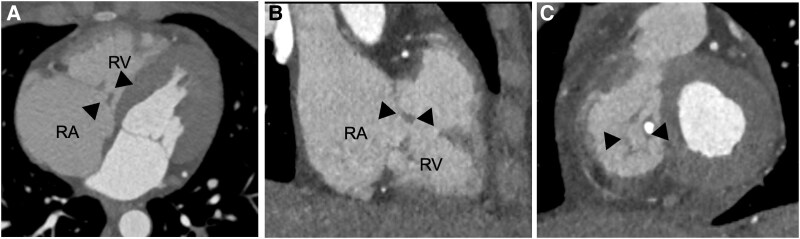
Cardiac computed tomography in four-chamber view (*A*), two-chamber view (*B*), and short-axis view (*C*). Dilation of the right-sided chambers, including the right atrium and right ventricle, is noted (*A*). The arrows indicate significant thickening of all three leaflets, with calcification of the septal leaflet and central coaptation defect. RA, right atrium; RV, right ventricle.

On transfer to our tertiary centre, she was afebrile but displayed jugular venous distension and a holosystolic murmur accentuated by inspiration. Repeat TTE confirmed right-sided volume overload and normal left ventricular ejection fraction (55%). Three-dimensional vena contracta area (3-D VCA) was 0.95 cm² (*Figure 1* and Supplementary material online, *Videos 1* and *2*), classifying the lesion as massive tricuspid regurgitation under the extended grading system of Utsunomiya *et al*. (massive ≥ 0.75–<1.0 cm²). Cardiac CT angiography excluded coronary disease and corroborated irregular thickening of the tricuspid leaflets (*Figure 2*).

Considering the recent SAH, transoesophageal echocardiography (TEE) was deferred. A Heart Team (cardiology, cardiac surgery, rheumatology, neurology) favoured LSE over infective endocarditis owing to sterile cultures, APS background, and imaging findings. Corticosteroids were tapered to physiologic dose and surgery was electively scheduled 21 days after the SAH, in accordance with current guidance to delay cardiopulmonary bypass 2–4 weeks after minor intracranial bleeding.

**Figure 3:**
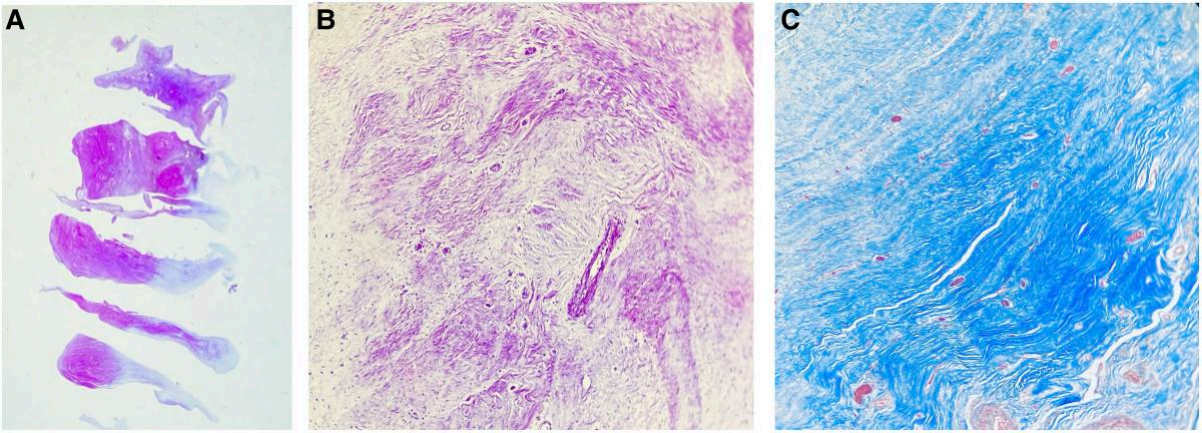
(*A*) Haematoxylin-eosin staining of the tricuspid valve. (*B*) Negative Gram stain showing no evidence of microorganisms. (*C*) Masson’s trichrome staining revealing fibrohyaline degenerative changes (blue deposits).

The patient underwent tricuspid valve replacement with a 31-mm bovine pericardial bioprosthesis. Cardiopulmonary bypass was conducted with standard heparinization (activated clotting time target ≈380 s) and intra-operative antifibrinolytic therapy (tranexamic acid). Inspection revealed friable vegetations on all three native leaflets and a 400-mL serous pericardial effusion. Histopathology confirmed sterile platelet–fibrin vegetations, consistent with LSE (*Figure 3*).

Post-operative recovery was uneventful; fever resolved and right-sided pressures normalized. Warfarin (target INR 2.5–3.5) was begun and bridged with unfractionated heparin. Persistent serological activity (high anti-dsDNA, low complement) at 1 month prompted B-cell depletion with rituximab plus hydroxychloroquine and tapering prednisone.

At 3-month follow-up, the patient was asymptomatic (NYHA class I). Transthoracic echocardiography showed a competent prosthetic valve with normal right-sided dimensions and no recurrent vegetations.

## Discussion

Libman–Sacks endocarditis is the most frequent valvular manifestation of SLE and results from endothelial injury with immune-complex deposition and sterile platelet–fibrin thrombi that can distort valve architecture and embolize. Although left-sided valves predominate, isolated tricuspid involvement is exceptional and has been documented mainly as case reports across the last decades (see *Table 1*). The clinical spectrum ranges from incidental murmur to right-sided heart failure, culture-negative fever, or embolic events and is observed predominantly in women with SLE/APS, consistent with our patient.^1–3,10^

**Table 1:** Published cases of isolated tricuspid Libman–Sacks endocarditis (1990–2025)

Author, year	Age/sex	APS	Clinical presentation	Echo severity	Management	Outcome
Mahajan 2017	34 F	Yes	Severe TR, dyspnoea	Torrential	TVR (bioprosthesis)	NYHA I at 12 mo
Unic 2017	60 F	Yes	Fever, emboli	Severe	TVR (mechanical)	NYHA II at 6 mo
Moaref 2010	29 F	No	Incidental murmur	Moderate	Medical	Stable at 3 yr
Zurick 2007	45 F	Yes	Right-HF/paradoxical embolism	Massive	Repair	Asymptomatic 18 mo
Migliorini 2022	38–45 F	Yes	Severe TR (pregnancy)	Torrential	TVR	NYHA I 6 mo
Vacca 2023	26 F	Yes	Acute presentation	Severe	TVR (not specified)	Good short-term
Bai 2015	20 F	SLE (±APS)	Acute RHF	Severe	TVR (bioprosthesis)	Good early

Distinguishing LSE from infective endocarditis is critical because management and prognosis diverge. In LSE, blood cultures are typically negative; vegetations tend to be sessile/heterogeneous and often multiple; and autoimmune/APS activity is common. Three-dimensional TEE (3-D TEE) improves detection of small lesions and defines their relationship to the subvalvular apparatus, while cardiac CT offers complementary anatomic information when TEE is deferred (e.g. after recent intracranial haemorrhage), as in this case.^1^

Two-dimensional measures (e.g. PISA-based EROA) may underestimate tricuspid regurgitation (TR) severity when the regurgitant orifice is noncircular and dynamic. Three-dimensional vena-contracta area (3-D VCA) correlates better with true regurgitant burden and has enabled extended grading (including ‘massive’ and ‘torrential’). In our patient, a 3-D VCA of 0.95 cm² reclassified the lesion as massive TR despite a modest 2-D EROA (30 mm²), supporting surgical referral.^4,11^

The 2021 ESC/EACTS and 2020 ACC/AHA guidelines support intervention for symptomatic isolated TR of massive or greater severity, irrespective of aetiology, with Heart Team deliberation to individualize timing and strategy—particularly when autoimmune and neurovascular issues coexist.^5,6^ In the present case, the combination of large right-sided vegetations, haemodynamically significant TR, and right-sided volume overload prompted surgical management once neurovascular risk was acceptable.

Tricuspid repair is preferable when feasible; however, in LSE, the diffuse leaflet thickening, friability, and destruction often preclude durable repair. Comparative evidence specific to tricuspid valve replacement (TVR) shows no survival advantage of mechanical over bioprosthetic valves at 30 days or long term, while highlighting the vulnerability of mechanical prostheses to valve-related events (thrombosis/embolism/bleeding) in the low-flow right heart. Contemporary bioprostheses demonstrate acceptable durability in this position. Even in patients who already require lifelong anticoagulation for APS, a bioprosthesis may mitigate tricuspid-position thrombosis risk while systemic anticoagulation is maintained for APS. Our decision reflected non-repairable anatomy, the lower thrombogenicity of bioprostheses at the tricuspid position, and the need for vitamin K antagonist (VKA) therapy for APS regardless of prosthesis type.^7,8^

In high-risk (triple-positive) APS, EULAR 2019 recommendations favour VKA—INR target 2–3 (or 3–4 in selected arterial events)—and advise against routine DOAC use. The TRAPS trial in triple-positive APS was stopped early due to excess events with rivaroxaban vs. warfarin. In the context of bioprosthetic TVR, valvular guidelines recommend VKA for at least the first 3 months; in our patient, APS warranted indefinite VKA irrespective of prosthesis type.^5,6,9,12^

Cardiac surgery with cardiopulmonary bypass (CPB) after recent intracranial haemorrhage carries a risk of rebleeding. Contemporary neuro–cardiac literature supports deferring ∼2–4 weeks when clinically feasible, guided by serial neuroimaging and meticulous intraoperative anticoagulation protocols. We delayed 21 days after a small, stable SAH, used standard heparinization (target ACT ≈ 380 s) and tranexamic acid, and observed no rebleeding—consistent with multicentre and systematic data.^13,14^

## Long-term immunomodulation and surveillance

Beyond the success of surgery, disease control is essential to reduce inflammatory activity and the risk of new valvular lesions. Corticosteroids, hydroxychloroquine, and B-cell depletion with rituximab have been associated with improved disease control and, in case series, lower recurrence. Given the possibility of subclinical recurrence, we recommend serial imaging (TTE ± 3-D/TEE) at 6–12 months to assess prosthetic integrity and screen for new lesions, along with lifelong VKA in APS.^1–3,10^

## Clinical implications

This case underscores three practical messages: (i) maintain a high index of suspicion for LSE in SLE/APS patients with culture-negative right-sided vegetations; (ii) use 3-D VCA to reconcile discrepant 2-D metrics and substantiate surgical indications; and (iii) after recent SAH, timed surgery with careful anticoagulation and selection of a bioprosthesis in the tricuspid position can achieve excellent outcomes when integrated with long-term anticoagulation and modern immunomodulation.

## Key take-home messages

Isolated tricuspid LSE should be considered in culture-negative right-sided endocarditis with autoimmune features; 3-D quantification can alter management; and integrated Heart Team care enables timely surgery, pragmatic selection of a bioprosthesis to minimize tricuspid-position thrombosis, lifelong VKA for APS, and sustained immunomodulation to prevent recurrence.

## Conclusion

Isolated tricuspid LSE is a rare but treatable entity. Early Heart Team evaluation, multimodality imaging and timely surgery—combined with tailored immunosuppression and lifelong anticoagulation—can achieve excellent functional recovery and prevent thromboembolic complications.

## Lead author biography


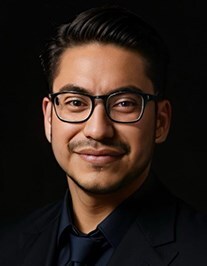


Dr Gamaliel Alejandro Velasquez-Orozco is a cardiology fellow at the National Medical Center ‘20 de Noviembre’, affiliated with the National Autonomous University of Mexico (UNAM). He completed his residency in internal medicine at Hospital General San Juan de Dios in Guatemala City, under the Universidad de San Carlos de Guatemala. His clinical and research interests include advanced heart failure, cardiac imaging, and coronary syndromes

## Supplementary Material

ytaf493_Supplementary_Data

## Data Availability

The data underlying this article are available in the article and in its online Supplementary material.
